# Interfacial Solar Vapour Generation: An Emerging Platform for Sustainable Clean Water Harvesting

**DOI:** 10.3390/molecules28155721

**Published:** 2023-07-28

**Authors:** Panpan Zhang, Wenjing Yuan

**Affiliations:** 1 School of Chemical Engineering and Technology, Hebei University of Technology, Tianjin 300130, China; 2 Engineering Research Center of Seawater Utilization of Ministry of Education, Tianjin 300130, China; 3 Hebei Collaborative Innovation Center of Modern Marine Chemical Technology, Tianjin 300130, China; 4 Tianjin Key Laboratory of Materials Laminating Fabrication and Interface Control Technology, School of Materials Science & Engineering, Hebei University of Technology, Tianjin 300130, China; yuanwenjing_07@126.com

Water is a precious resource of paramount importance on Earth, playing a critical role in the evolution of life [1]. Despite water covering approximately 71% of the Earth’s surface, saline water constitutes 97.5% of the total. Out of the remaining 2.5% of freshwater resources, merely 0.03% of the world’s total water is renewable freshwater directly accessible for human consumption [2]. Reports and studies conducted by the World Health Organization reveal that severe water scarcity affects 80 countries and regions worldwide, with over 55% of the global population facing a shortage of water resources [3]. Conventional water treatment technologies, including thermal distillation, adsorption filtration, catalytic degradation, and membrane techniques, suffer from high energy consumption, expensive equipment investment, low energy utilisation efficiency, and inadequate production of clean water [4,5]. As such, there is an urgent need to develop economical, efficient, and sustainable water purification and recycling technologies to alleviate the pressing issue of water scarcity.

Interfacial solar vapour generation (ISVG) systems effectively absorb and convert solar radiation into heat, concentrating the heat at the interface where evaporation takes place. This leads to a substantial enhancement in the solar water evaporation rate, offering effective, affordable, and off-grid access to recover impaired and unconventional water that is critical to a sustainable, safe, and potable water supply [6,7,8,9]. Currently, continuous efforts are being devoted to advancing ISVG systems through rational material/structural engineering of solar evaporators and solar distillers for high-output clean water harvesting. The regulation strategies are composed of light absorption, heat localisation, extra energy harvesting, and reduced evaporation enthalpy, all directed towards achieving efficient solar-to-thermal conversion [10,11,12,13]. Additionally, practical applications are being explored, encompassing water purification from seawater and complex wastewaters, as well as the development of diverse prototype solar distillers for water collection in natural environments [14,15,16].

Despite the rapid developments and significant improvements made in ISVG systems, with solar thermal conversion efficiency approaching 100%, the practical applications of ISVG still face challenges. Specifically, long-term fouling resistance and high stability towards complex wastewaters remain major concerns. Furthermore, the final water yield of solar distillers is limited by slow water condensation, ineffective heat transfer, and low energy efficiency. In this regard, we highlight several challenges and opportunities to substantially advance ISVG-based water purification for addressing clean energy needs and water scarcity issues.

(1) Constructing novel-type solar evaporators for high-rate water evaporation: Solar evaporators should be designed with a comprehensive consideration of low cost, scalability, stability, and adaptability to enable their practical application in addressing water scarcity. To achieve this, strategies may include reducing the cost of solar evaporators, simplifying the preparation process, and enhancing long-term stability for use in harsh environments (e.g., strong mechanical stability, resistance to pollutants, etc.).
(2) Upgrading the configuration of solar distillers for high-yield water harvesting: Under prolonged sunlight exposure, the high temperature and humidity inside solar distillers, coupled with inefficient heat transfer at the condensation interface, hinder rapid and effective water vapor condensation and latent heat recovery, resulting in clean water collection yields below 50%. To address this challenge, optimisation of the solar distiller’s exterior design can enhance sunlight utilisation; modification of the condenser’s surface microstructure and chemical properties can promote water droplet condensation; and improvements in heat and mass transfer can effectively manage thermal energy. Furthermore, the next generation of solar distillers should prioritise considerations of stability, cost, and maintenance as core issues.
(3) Deeper understanding of the underlying mechanisms in the ISVG system: An in-depth understanding of key factors, such as efficient photothermal conversion, light absorption, water transport, and heat transfer processes, is essential for improving energy utilisation efficiency. Although some studies are gradually focusing on the interaction between materials and water and the great influence of this interaction on the evaporation behaviour of water, these studies are still in their infancy, and the core steps and related mechanisms are not clearly elaborated. For example, precise modelling or characterisation of the interaction of surface functional groups with water molecules has not yet been established, and there is a lack of unity between energy transfer processes and evaporation mechanisms on the microscopic scale involving mass transport, fluid dynamics, heat transfer, and interactions between protons, phonons, electrons, molecules, and ions.
(4) Promoting the practical application of ISVG-based clean water collection: For the field of ISVG systems, which has been explored for sustainable clean water harvesting, more external practical explorations should be initiated, including the evaluation of multiple dimensions such as cost, environmental impact, and substitution of existing technologies, so as to advance the practical application of interfacial evaporation technology. More unremitting efforts and research are needed to solve the above key issues.

This emerging ISVG technology for water purification fits within the scope of the Special Issue entitled “Advanced Materials for Energy Conversion and Water Sustainability”, which holds the potential to enhance thermal utilisation efficiency, thereby playing a positive role in addressing real-world issues such as the energy crisis and water scarcity.

Finally, we anticipate that more intriguing findings and research associated with this Special Issue can be published in *Molecules*. These contributions will provide valuable guidelines for advancing low energy consumption and environmentally friendly approaches to clean water harvesting.
